# Clinical Symptoms and Outcomes of Severe Pneumonia Caused by *Chlamydia psittaci* in Southwest China

**DOI:** 10.3389/fcimb.2021.727594

**Published:** 2022-01-06

**Authors:** Fuxun Yang, Jiajia Li, Bo Qi, Longfei Zou, Zongming Shi, Yu Lei, Jun Li, Xiaoxiu Luo, Fan Zeng, Sen Lu, Xiaobo Huang, Rongan Liu, Yunping Lan

**Affiliations:** ^1^ Department of ICU, Sichuan Provincial People’s Hospital, University of Electronic Science and Technology of China, Chengdu, China; ^2^ Department of Intensive Care Unit, 903 Hospital, Mianyang, China; ^3^ Department of ICU, Affiliated Hospital of Southwest Medical University, Luzhou, China; ^4^ Department of ICU, Neijiang People’s Hospital Southwest Medical University, Sichuan, China; ^5^ Department of ICU, Shifang City People’s Hospital Affiliated to North Sichuan Medical College, Shifang, China

**Keywords:** *Chlamydia psittaci*, pneumonia, clinical symptoms, mNGS, ICU

## Abstract

Here, we aimed to retrospectively analyze the clinical characteristics of 27 patients with severe pneumonia caused by *Chlamydia psittaci* between January 2019 and April 2021 in southwest China. To this end, we collected data on the exposure history, clinical symptoms, laboratory examination, imaging characteristics, evolution, etiology, treatment, and outcomes to suggest a better diagnosis and prevention system. Our results showed that a metagenomic next-generation sequencing test could provide early diagnosis. All patients were sensitive to quinolones and tetracyclines, and the recovery rate was relatively high. Overall, all patients were in critical condition with moderate to severe acute respiratory distress syndrome and shock. In conclusion, early diagnosis of pneumonia caused by *C. psittaci* depends on effective molecular testing, and most patients recover after treatment.

## 1 Introduction

*Chlamydia psittaci* is a gram-negative bacterium with a size between bacteria and viruses. It widely exists in wild birds or poultry such as parrots, wild pigeons, chickens, and ducks. Ten genotypes are known that correspond to different hosts and virulence, causing zoonotic infectious diseases (Knittler and Sachse, 2015). Humans are usually infected by inhaling the organisms in dry feces or bird feather dust when caged birds spread their wings. Cage cleaning may pose a risk of infection. Bird bites, mouth to beak contact, and even brief contact, such as visiting bird parks, are also related to the spread of this infection (Hinton et al., 1993). The typical clinical symptoms include fever, chills, headache, dry cough, gastrointestinal problems, severe pneumonia, endocarditis, jaundice, and nervous system complications (Johnston et al., 2000; Hogerwerf et al., 2017; Rybarczyk et al., 2019). There are only a few reports that of clustered cases in families and hospitals, indicating the possibility of human-to-human transmission (Wallensten et al., 2014). *C. psittaci* pneumonia has been recognized worldwide, including in the United States, United Kingdom, Europe, Middle East, and Australia, and was previously thought to cause 1% of the cases of community-acquired pneumonia (Petrovay and Balla, 2008). However, the exact incidence and prevalence of psittacosis are difficult to determine or may even be underestimated owing to the lack of routine tests and the differences in sensitivity and specificity of common diagnostic tests (Rybarczyk et al., 2020). Pneumonia caused by *C. psittaci* usually has mild symptoms, but some patients can progress to severe disease and even die of respiratory failure and multiple organ failure (Branley et al., 2014). For critically ill patients, successful treatment depends on accurate diagnosis and appropriate support. With the rapid development of molecular methods, especially metagenomic next generation sequencing (mNGS), the early diagnosis of diseases is possible, and the number of related cases reported has increased (Shi et al., 2021; Teng et al., 2021; Yuan et al., 2021). In this study, we summarize the epidemiology, clinical characteristics, laboratory data, and clinical outcomes of 27 cases of severe pneumonia caused by C. *psittaci* diagnosed by mNGS, to provide a basis for effective diagnosis and treatment.

## 2 Methods

### 2.1 Setting

This study was approved by the Ethics Committee of Sichuan Provincial People’s Hospital (No. 2020-111), and all data were anonymous before analysis. The study was carried out in accordance with the Declaration of Helsinki. The data of the study were obtained from the comprehensive ICU of four tertiary A hospitals in southwest China, with more than 280 ICU beds in total.

### 2.2 Study Design

In this retrospective study, we included 27 cases of severe *C. psittaci* pneumonia diagnosed by mNGS in the ICU of four tertiary A hospitals in southwest China from May 2018 to March 2020. The diagnostic criteria of *C. psittaci* pneumonia refer to previous literature reports (Su et al., 2021). We recorded data on age, sex, exposure history, comorbidities, onset course, symptoms, signs, imaging, laboratory examination, method of diagnosis, treatment, and clinical outcomes.

### 2.3 mNGS Detection Method

A 1.5–3 mL bronchoalveolar lavage fluid (BALF) sample from each patient was collected according to standard procedures. A 1.5 mL microcentrifuge tube with 0.6 mL sample, enzyme, and 1 g 0.5 mm glass bead were attached to a horizontal platform on a vortex mixer and agitated vigorously at 2800–3200 rpm for 30 min. A 0.3 mL sample was separated into a new 1.5 mL microcentrifuge tube and DNA was extracted using the TIANamp Micro DNA Kit (DP316, TIANGEN BIOTECH) according to the manufacturer’s recommendation. Then, DNA libraries were constructed through DNA-fragmentation, end-repair, adapter-ligation, and PCR amplification. Agilent 2100 was used for quality control of the DNA libraries. Quality qualified libraries were sequenced by BGISEQ-50/MGISEQ-2000 platform (Jeon et al., 2014). High-quality sequencing data were generated by removing low-quality reads, followed by computational subtraction of human host sequences mapped to the human reference genome (hg19) using Burrows–Wheeler Alignment (Li and Durbin, 2009) The remaining data, by removal of low-complexity reads, were classified by simultaneously aligning to four Microbial Genome Databases, consisting of bacteria, fungi, viruses, and parasites.

The classification reference databases were downloaded from NCBI (ftp://ftp.ncbi.nlm.nih.gov/genomes/). RefSeq contained 4,945 whole genome sequences of viral taxa, 6,350 bacterial genomes or scaffolds, 1,064 fungi related to human infection, and 234 parasites associated with human diseases.

### 2.4 Statistical Method

Statistical analyses were performed using R software version 3.5.1 (R Foundation for Statistical Computing, Vienna, Austria) and SPSS version 20.0 (IBM Corporation, Armonk, NY). Categorical data were summarized using frequencies and percentages. Continuous data were summarized using median and interquartile range (IQR). Unpaired Wilcoxon’s rank-sum test was used to test differences for continuous variables and Fisher’s exact test was used for categorical variables. Survival rate was calculated and plotted by Kaplan-Meier method, and univariate analysis was performed by log-rank test. All reported p-values were two-sided and statistical significance was set at p < 0.05.

## 3 Results

### 3.1 Epidemiology

The average age of the patients was 60 years (35–87 years). One woman was in her third trimester. Except for four patients who kept pigeons and six who had contact with poultry in open markets, the remaining patients had no clear history of exposure to birds (**Table 1**).

**Table 1:** Basic characteristics of patients (n = 27) included in the study.

	All (N=27)	survivor (N=23)	Non-survivor (N=4)	P
gender, male, n (%)	19 (70.4%)	17 (73.9%)	2 (50.0%)	0.334
Age, Median [Min, Max]	60.0 [35.0, 87.0]	60.0 [35.0, 87.0]	67.0 [54.0, 83.0]	0.895
Exposure to bird, n (%)	10 (37.0%)	9 (39.1%)	1 (25.0%)	0.594
Time from onset to diagnosis, Median [Min, Max]	11.0 [2.00, 20.0]	10.0 [2.00, 20.0]	13.0 [9.00, 17.0]	0.501
Time from onset to respiratory failure, Median [Min, Max]	7.00 [1.00, 16.0]	7.0[1.0,16.0]	6.5 [6.0,14.0]	0.896
Admission symptoms				
Cough, n (%)	19 (70.4%)	16 (69.6%)	3 (75.0%)	0.826
Backache, n (%)	1 (3.7%)	1 (4.3%)	0 (0%)	0.852
fever, n (%)	23 (85.2%)	20 (87.0%)	3 (75.0%)	0.542
dyspnea, n (%)	22 (81.5%)	19 (82.6%)	3 (75.0%)	0.719
headache, n (%)	10 (37.0%)	8 (34.8%)	2 (50.0%)	0.565
diarrhea, n (%)	3 (11.1%)	3 (13.0%)	0 (0%)	0.605
Characteristics at ICU admission				
APACHE II, Median [Min, Max]	16.0 [8.00, 35.0]	15.0 [8.00, 35.0]	20.0 [17.0, 26.0]	0.411
≤10	6 (22.2%)	6 (26.1%)	0	0.251
10-15	4 (14.8%)	4 (17.4%)	0	
>15	17 (63.0%)	13 (56.5)	4 (100%)	
SOFA, Median [Min, Max]	5.00 [2.00, 9.00]	5.00 [2.00, 9.00]	8.00 [6.00, 8.00]	0.199
PaO2/FIO2 ratio, mm Hg, Median [Min, Max]	162 [46.0, 248]	166 [46.0, 248]	99.0 [69.0, 135]	0.393
>200	5 (18.5%)	5 (21.7%)	0	0.277
100-200	16 (59.3%)	14 (60.9%)	2 (50%)	
<100	6 (22.2%)	4 (17.4%)	2 (50%)	

CI, confidence interval; OR, odds ratio.

### 3.2 History and Clinical Manifestations

Among the 27 patients, 10 had hypertension, 11 had cirrhosis, one had diabetes, one had pneumoconiosis, and one had gout. The symptoms of most patients were high fever (≥39°C), cough with or without expectoration, dizziness, headache, and disturbance of consciousness (**Table 1**). The median time from admission to diagnosis was 11 days (2–20 days), and the median time from admission to respiratory failure was 11 days (1–16 days). Eleven patients (40.7%) developed sepsis and acute respiratory failure upon admission to the intensive care unit (ICU).

### 3.3 Auxiliary Examination

#### 3.3.1 Laboratory Tests

All patients were tested for *Chlamydia trachomatis* IgM on admission, and eight were positive (**Table 2**). Most patients had normal white blood cells but elevated hs-CRP(high-sensitivity C-reactive protein ), lymphocytopenia, hyponatremia, and abnormal liver enzymes. The APTT, D-Dimer, MYO and HS-TNTI of the death group and the survival group were statistically significant.

**Table 2:** Laboratory test.

Laboratory findings	All (N=27)	survivor (N=23)	Non-survivor (N=4)	P
procalcitonin, Median [Min, Max]	1.19 [0.05, 7.23]	1.15 [0.05, 7.23]	2.46 [0.27, 5.72]	0.830
≤0.05	1 (14.8%)	1 (4.3%)	0	0.909
0.05-0.50	7 (25.9%)	6 (26.1%)	1 (25%)	
>0.50	19 (59.3%)	16 (69.6%)	3 (75%)	
IgM positive, n (%)	8 (29.6%)	6 (26.1%)	2 (50.0%)	0.347
Leucocyte, ×10⁹/L, Median [Min, Max]	8.71 [3.65, 24.3]	8.91 [3.90, 24.3]	6.84 [3.65, 11.3]	0.549
<4	2 (7.4%)	1 (4.4%)	1 (25%)	0.346
4-10	17 (63.0%)	15 (65.2%)	2 (50%)	
>10	8 (29.6%)	7 (30.4%)	1 (25%)	
Lymphocyte, × 10⁹/L, Median [Min, Max]	0.56 [0.12, 2.03]	0.520[0.12, 2.03]	0.67 [0.34, 0.86]	0.251
>0.8	4 (14.8%)	3 (13.0%)	1 (25%)	0.315
0.6-0.8	7 (25.9%)	5 (21.7%)	2 (50%)	
<0.6	16 (59.3%)	15 (65.2%)	1 (25%)	
Platelets, × 10⁹/L, Median [Min, Max]	155 [63.0, 350]	159 [63.0, 350]	129 [109, 166]	0.056
<100× 10⁹/L	4 (11.0%)	3 (13.0%)	0	0.444
Hemoglobin, g/dL, Median [Min, Max]	127[72,153]]	122[72,153]	134[122,137]	0.251
Anemia	4 (14.8%)	4 (17.4%)	0	0.366
CRP, Median [Min, Max]	153 [33.7, 274]	153 [33.7, 265]	169 [123, 274]	0.921
>100	23 (85.2%)	19 (82.6%)	4 (100%)	
Albumin (g L-1), Median [Min, Max]	29.7 [24.1, 44.7]	29.7 [24.1, 44.7]	34.4 [27.2, 40.3]	0.562
<30	14 (48.2%)	12 (47.8%)	2 (50%)	0.936
bilirubin (g L-1), Median [Min, Max]	19.0 [8.60, 69.9]	20.4 [8.60, 69.9]	18.6 [11.9, 19.1]	0.418
>20	12 (55.6%)	12 (47.8%)	0	0.053
APTT (s), Median [Min, Max]	33.4 [26.4, 70.0]	32.2 [26.4, 70.0]	43.8 [27.4, 55.5]	0.108
>40	7 (25.9%)	4 (17.39%)	3 (75%)	0.015
D-dimer (mg/l), Median [Min, Max]	4.31 [0.88, 38.6]	4.31 [0.88, 38.6]	4.33 [2.79, 17.50]	0.019
<1.0	2 (7.4%)	2 (8.7%)	0	0.511
1.0-2.0	4 (14.8%)	4 (17.4%)	0	
>2.0	21 (77.8%)	17 (73.9%)	4 (100%)	
CK-MB (ng/ml)	1.20 [0.30, 23.4]	1.00 [0.30, 20.7]	9.00 [1.40, 23.4]	
>2.0	6 (22.2%)	4 (17.4%)	2 (50%)	0.148
MYO (ng/ml)	121 [17.0, 35400]	81.4 [17.0, 2250]	448 [21.7, 35400]	
>300	7 (25.9%)	4 (17.4%)	3 (75%)	0.015
HS-TNTI (ng/L)	18.9 [0.260, 130]	18.9 [0.260, 130]	78.9 [22.6, 129]	
>50	6 (22.2%)	3 (13.0%)	3 (75%)	0.006
mNGS-results	152 [7.00, 5350]	4530 [337, 15200]	337 [7.00, 15200]	
>1000	9 (33.3%)	6 (26.1%)	3 (75%)	0.055

ECMO, extracorporeal membrane oxygenation; MV, mechanical ventilation; CRRT, continuous renal replacement therapy; PCT, procalcitonin; CRP, C-reactive protein.

#### 3.3.2 Imaging Examination

**Figure 1:**
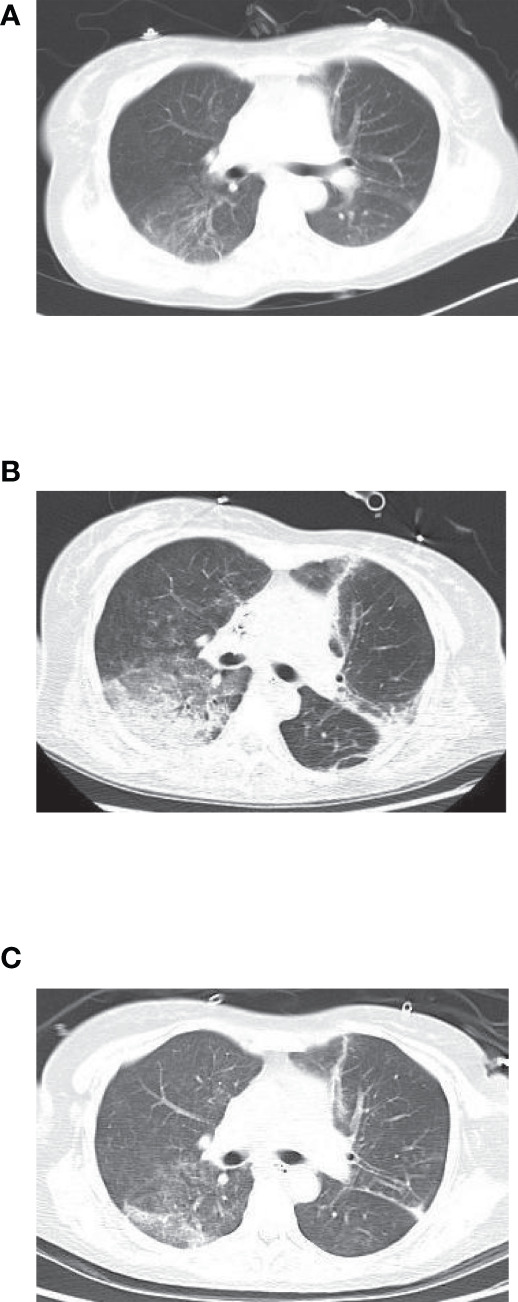
**(A)** Before treatment. **(B)** Before metagenomic next-generation sequencing (mNGS). **(C)** After the treatment.

Chest imaging showed that early lesions were mainly involved in a single lobe of the right lung, with inflammatory exudation and local consolidation. As the disease progressed, inflammatory exudates developed in both lobes with pleural effusion (**Figures 1A–C**). One patient was discharged with residual pulmonary interstitial changes, and the rest had absorbed inflammatory pleural exudate.

#### 3.3.3 mNGS Results

All patients underwent fibrobronchoscopy, and they were tested based on BALF, mNGS, and routine microbiology within 3 days of admission to the ICU. mNGS using BALF samples showed the presence of *Helicobacillus* (7–15234 copies) (**Supplementary Material**) in all patients, *Candida albicans* in six patients, *Corynebacterium striatum* in three patients, and *Streptococcus pneumoniae* in two patients. mNGS testing of blood samples from five patients revealed the presence of *Chlamydia psittaci*.

### 3.4 Treatment Process

All patients were treated with quinolones, macrolides, or tetracycline antibiotics. Among them, 15 patients were treated with class III drugs before diagnosis (14 quinolones, six levofloxacin, eight moxifloxacin; Macrolide 1 (azithromycin 1)). After diagnosis, 27 patients had their medication adjusted accordingly as follows: 17 cases (63%) were quinolones (levofloxacin: six cases, moxifloxacin: eight cases); there were six cases of tetracycline treatment, accounting for 22.2% (one case of minocycline, five cases of doxycycline), and five cases of macrolide treatment, accounting for 18.5% (five cases of azithromycin). One patient received a combination of azithromycin and doxycycline. Among the 27 patients, 21 patients had fever. The duration of fever withdrawal was approximately 1–4 d, with an average of 2.3 d, accompanied by a significant decrease in hs-CRP, and the effect was significant. Five patients were treated with methylprednisolone (80–320 mg, bid) for inflammatory exudation of the chest early in the disease and did not show spread of infection. Six patients (22.2%) developed nosocomial infections, and one patient (3.7%) had carbapenem-resistant *Klebsiella pneumoniae* and carbapenem-resistant *Acinetobacter baumannii* in the lungs and blood, increasing the risk of death.

Among the 27 patients, five patients had mild acute respiratory distress syndrome (ARDS), 16 patients had moderate ARDS, and six patients had severe ARDS (**Table 3**). All patients received mechanical ventilation, including 14 patients with invasive mechanical ventilation and 23 patients with non-invasive mechanical ventilation. In 10 patients, it was difficult to maintain oxygenation during the treatment of non-invasive ventilator-assisted ventilation, and thus, treatment was replaced with invasive mechanical ventilation. All patients receiving invasive mechanical ventilation were treated with a small tidal volume protective pulmonary ventilation, sedation and analgesia, and prone ventilation as recommended in ARDS treatment guidelines. Three patients with severe ARDS developed extremely severe ARDS during the course of 8–12 d and were given intravenous extracorporeal membrane oxygenation for 6, 10 and 16 d, respectively. In the course of treatment, 4 patients were treated with CRRT because of renal function injury, of which 3 cases died and 1 patient survived and discharged without residual renal function injury.

**Table 3:** Treatment and outcomes.

	All (N=27)	survivor (N=23)	Non-survivor (N=4)	P
MV, n (%)	11 (40.7%)	7 (30.4%)	4 (100.0%)	0.019
Intubation days, Median [Min, Max]	6.00 [1.00, 17.0]	6.00 [1.00, 17.0]	4.50 [3.00, 10.0]	0.775
ECMO, n (%)	3 (11.1%)	3 (13.0%)	0 (0%)	0.605
CRRT, n (%)	4 (14.8%)	2 (50%)	2 (8.7%)	0.032

MV, Mechanical ventilation; ECMO, extracorporeal membrane oxygenation; CRRT, continuous renal replacement therapy.

### 3.5 Clinical Outcomes

Overall, the majority of patients had a good prognosis (**Table 4**). Of the 27 patients, 23 patients survived, and one patient had residual pulmonary interstitial changes without significant decreased activity tolerance. None of the surviving patients had serious complications during follow-up. Four patients died from secondary nosocomial infections. The vital signs of three patients were not maintained after intravenous extracorporeal membrane oxygenation support therapy, including one pregnant woman. All three patients recovered well and were discharged easily. No complications occurred in the follow-up of 3 months. In the case of the pregnant woman, after admission, even though we carried out routine fetal monitoring of the patient, death of the fetus occurred due to various factors, such as severe hypoxia, antimicrobial agents, sedation and analgesia, medical treatment, and the use of anticoagulant drugs, and 2 d after tracheal intubation, labor was induced with the informed consent of the patient’s family.

**Table 4:** Univariable logistic regression of factors associated with death risk of critically ill patients with *Chlamydia psittaci*.

	OR	95%CI	P
Age≥65,years	1.06	(0.96,1.16)	0.248
Exposure to bird	0.51	(0.05,5.80)	0.594
Time from onset to diagnosis, days	1.15	(0.87,1.50)	0.321
Time from onset to respiratory failure, days	1.05	(0.79,1.04)	0.722
PCT≥0.5,ng/dl	1.31	(0.12,14.93)	0.826
APACHE II ≥ 25	7.33	(0.36,50.71)	0.196
SOFA	2.05	(0.94,4.46)	0.07
IgM positive	2.83	(0.32,24.81)	0.347
PaO2/FIO2 ratio >100 mmHg	0.21	(0.02,1.97)	0.172
WBC < 4 × 10⁹/L	0.14	(0.01,2.87)	0.196
Lym < 0.6 × 10⁹/L	5.62	(0.50,63.60)	0.162
Albumin < 30 g L-1	1.09	(0.13,9.12)	0.936
bilirubin ≥20 g L-1	0.94	(0.81,1.09)	0.417
APTT ≥40 s	14.25	(1.16,174.8)	0.038
CK-MB ≥ 2ng/ml	4.75	(0.51,44.5)	0.172
MYO ≥ 300ng/ml	14.25	(1.16,74.8)	0.038
HS-TNTI ≥ 50ng/l	20.0	(1.53,60.7)	0.022
mNGS-results ≥ 1000	8.5	(0.74,98.2)	0.087
Intubation days	0.89	(0.62,1.29)	0.536
CRRT	10.5	(0.92,120.25)	0.059

SOFA, Sequential Organ Failure Assessment.

In the pregnant woman’s case, even though we conducted regular fetal monitoring after the patient was admitted to hospital, owing to various factors such as severe hypoxia, anti-infective drugs, sedation and analgesia, and the use of anticoagulant drugs after ECMO, fetal death was found *in utero*, 2 days after endotracheal intubation, and labor was induced with the informed consent of the patient’s family. Univariate logistic regression of death factors in the study cohort showed that APTT ≥40 s, Myoglobin(MYO) ≥ 300ng/mL and Hypersensitive troponin I(HS-TNTI) ≥ 50ng/L were risk factors for death in severe patients (**Table 3**).

## 4 Discussion

As previously reported, nearly 63.7% of patients with *C. psittaci* infection are male (Yung and Grayson, 1988), which was in agreement with the rate in our study (69% male).The majority of infections reported in previous studies were in adults between the ages of 30 and 60 years (Shi et al., 2021), as was the case in our cohort. Usually, 90% of the patients have a history of exposure to poultry (Saeki et al., 1996), but in our study, only 31% of the patients reported a contact. Our analysis showed that fever was the most common symptom in patients—results that were consistent with those reported in a previous study (Yung and Grayson, 1988). Besides, nine patients showed neurological symptoms, such as disturbance of consciousness or confusion, whereas 19 patients were diagnosed with hyponatremia. Although the effects of psittacosis on the nervous system were first reported in 1880, the underlying mechanism remains unclear (Macfarlane and Macrae, 1983; Saeki et al., 1996). Four potential pathogenic mechanisms have been proposed: direct invasion of *C. psittaci* to the central nervous system, autoimmune mechanisms, embolism, and electrolyte disorders (Al-Kawi and Madkour, 1986; Vassallo and Shepherd, 1997; Walder et al., 2003). In a previously reported case of encephalopathy (Walder et al., 2003), *C. psittaci* was found in the cerebrospinal fluid of the patient, which may show that the first mechanism is the most possible. Since quinolones or tetracyclines are not able to cross the blood–brain barrier, the prognosis for patients with severe nervous system symptoms is often poor. Indeed, three of the four patients that died in the duration of our study developed neurological symptoms, despite the administration of antibiotics.

All patients showed markedly increased levels of hs-CRP and slightly increased levels of liver enzymes. A previous study reported that *C. psittaci* is more pathogenic and propagates faster than other *Chlamydia* species, causing severe inflammation. Therefore, the increase in white blood cells was not obvious at the early stages of the disease, but that of hs-CRP was rapid, and the results were consistent with those presented in a previous study (Knittler and Sachse, 2015). *C. psittaci* first enters the reticular endothelial cells of the liver and spleen to proliferate after inhalation through the respiratory tract, and consequently, patients with respiratory symptoms also show changes in liver enzymes. A previous study suggested that the diagnosis of *C. psittaci* should be considered when patients aged <60 years with fever, liver enzyme changes, or nervous system symptoms are admitted from early May to early October (Raeven et al., 2016), even if they have no history of contact with birds, to prevent septic shock and ARDS at a later stage.

We found that lymphocytopenia occurred in 93% of critically ill patients. Lymphocytopenia is a significant symptom of patients with severe community-acquired infections. It has been reported that some atypical types of pneumonia can destroy the cytoplasmic components of lymphocytes, leading to lymphocyte apoptosis (Gu et al., 2005). Lymphocytopenia is also common in critically ill patients with psittacosis. Indeed, the four patients that died showed a continuous decrease in the lymphocyte count throughout the course of the disease that was irreversible. Thus, further research is needed to explore the causes of lymphocytopenia caused by *C. psittaci*.

In addition, the increase of MYO and Hs-TNTi caused by chlamydia psittaci was also common in our patients, especially in patients who died. MYO≥300ng/mL and HS-TNTi ≥50ng/L were one of the high-risk death factors in patients with chlamydia psittaci pneumonia. In previous reports, the increase of (Su et al., 2021) CK was a high risk factor for severe chlamydia psittaci pneumonia. Our study further proved that not only CK, MYO and HS-TNTI related to myocardial enzymes were also high risk factors for severe patients, suggesting that myocardial injury caused by Chlamydia psittaci is not rare in clinic. In the clinical treatment, we should be highly alert to such patients to avoid severe cases.

Mechanical ventilation is the primary supportive treatment for critically ill patients. In the present study, all patients were provided oxygen therapy (PaO_2_/FiO_2_ < 300), whereas six patients with severe ARDS received lung-protective ventilation and prone position ventilation. Three of the latter also received ECMO adjuvant therapy.

A previous study showed that patients with psittacosis had a good prognosis after timely diagnosis and antibiotic treatment. We found that more than 50% of the patients (15/27 cases) were administered quinolones as a preliminary antibiotic treatment that was adjusted after the diagnosis based on the symptoms (Chen et al., 2020). Tetracycline antibiotics are the primary recommendation for *C. psittaci* pneumonia. In our cohort, all patients treated with tetracycline antibiotics were sensitive. However, presently tetracycline resistance is also one of the problems that need to be paid attention in a clinical setting. Tetracycline resistance originated from the environment (Thaker et al., 2010) but is now widely distributed in symbiotic bacteria and pathogenic bacteria, so has been also looking for a solution, clinical if use dehydration tetracycline or analogues inhibiting Tet (X7) (Gasparrini et al., 2020) can prevent the tetracycline antibiotics degradation and save efficacy or develop feasible adjuvant method to against tetracycline resistance by enzymatic inactivated (Markley et al., 2019). Therefore, in patients with severe disease who show tetracycline resistance, we may need to combine macrolides or quinolones. Previous studies showed that quinolones are not effective in patients with psittacosis. In our study, only four patients were treated with quinolones until recovery, whereas the remaining patients were quickly switched to macrolides or tetracyclines after the diagnosis of psittacosis. Most patients showed good efficacy and quick recovery. The four patients that died were administered carbapenems during the preliminary antibiotic treatment, although their procalcitonin was slightly increased at admission. The prolonged use of carbapenems led to multiple drug-resistant bacterial infections, followed by multiple organ failure and death.

Although systemic sex hormone therapy is controversial in ARDS patients, considering severe pneumonia and severe systemic inflammatory reaction, glucocorticoids were used in all patients, showing good clinical results without any complications. However, additional research and analysis of a higher number of cases is needed to confirm the results.

Clinical diagnosis of *C. psittaci* pneumonia is mainly based on the combination of clinical manifestations and laboratory examination. This theoretical gold standard method of pathogen culture is not routinely tested in most laboratories owing to the infectious nature of *C. psittaci* itself, which requires high levels of biosafety. Currently, commercially available laboratory tests mainly include serological, microbial culture, and nucleic acid amplification tests. The most commonly used serological tests include a complement binding test (CF) and microimmunofluorescent antibody test. The microimmunofluorescent antibody test is the most sensitive and specific serological test for *C. psittaci*, but it is only available in specialized laboratories (Wreghitt, 1993) The CF test is a test traditionally used for diagnosis and requires showing antibody titers at least four times higher than the upper normal limit in repeated serum samples. It has disadvantage of requiring repeated CF tests after 2 weeks and being unable to distinguish between different *Chlamydia* species. Early effective tetracycline therapy might also delay or attenuate the antibody response in critically ill patients. In addition, direct immunofluorescent antibody staining of respiratory secretions (sputum, throat swabs) has been used for rapid diagnosis (Oldach et al., 1993), but more studies are needed to demonstrate its sensitivity and specificity. As a result, molecular approaches are becoming popular. Nucleic acid amplification tests are considered more sensitive and specific than serological antibody tests. Unfortunately, this approach has not been carried out on a clinical scale and has only been conducted in cases of suspected *C. psittaci* outbreaks. With the development of mNGS, a variety of pathogens in different specimens can be quickly and accurately detected, including atypical pathogens, viruses, and fungi, among others, which are difficult to culture. It has the advantage of a wide detection range and no requirement for identifying suspected pathogens in advance and can be used for the diagnosis of lower respiratory tract infections. When treating patients with severe pneumonia, early identification of the pathogen is of utmost importance. With mNGS, etiological results can be obtained within 24 h at the earliest, and tetracycline-based antimicrobial therapy can be adjusted in time to minimize the time required to diagnose psittacosis and the disease process. At the same time, no other pathogens were detected by mNGS, thus reducing the use of unnecessary antibacterial drugs (such as carbapenems and antifungal drugs), effectively reducing the cost and time of hospitalization, and avoiding the emergence of drug-resistant bacteria. Several hospitals in this study were class-A tertiary teaching hospitals, but they only tested for *Chlamydia* IgM. Therefore, in this study, the application of Acer sequencing technology for the diagnosis of *C. psittaci* pneumonia could be used to quickly and specifically identify this species, thus shortening the diagnosis time, identifying other potential pathogens, and allowing for the initiation of targeted antimicrobial therapy in time.

This study had some limitations: 1) data were limited since the number of seriously ill patients was relatively small and 2) the CDC recommends a single IgM antibody titer of 1:16 or higher as a diagnostic test, but unfortunately, our institution does not have this requirement. *C. psittaci* IgM antibody was not detected in any of the patients. 3) Further studies with larger sample sizes are needed to support and confirm our findings.

Overall, for fever of unknown cause combined with exposure history, liver enzymology changes, and nervous system changes, we should be alert to the occurrence of *C. psittaci* pneumonia. Medication should be administered in a timely manner to avoid the later development of ARDS or sepsis or even multiple organ failure. Among all diagnostic methods, mNGS allows for early diagnosis that will help devise effective treatment plans and reduce the recovery time.

## Data Availability Statement

The original contributions presented in the study are included in the article/**Supplementary Material**. Further inquiries can be directed to the corresponding authors.

## Ethics Statement

The studies involving human participants were reviewed and approved by Sichuan Academy of Medical Sciences and Sichuan Provincial People’s Hospital Medical Ethics Committee. Written informed consent for participation was not required for this study in accordance with the national legislation and the institutional requirements.

## Author Contributions

FY and FZ wrote the original draft. LZ, ZS, and JuL provided some of the data. XL, RL, YLe, and SL undertook validation, writing, review, and editing. XH, JiL, and YLa undertook review and editing. BQ is responsible for the statistics of the data and the revision of the article. All authors contributed to the article and approved the submitted version.

## Funding

Funding was provided by the Key research and development project of Science and Technology of Sichuan Province (Grant no. 20ZDYF1870).

## Conflict of Interest

The authors declare that the research was conducted in the absence of any commercial or financial relationships that could be construed as a potential conflict of interest.

## Publisher’s Note

All claims expressed in this article are solely those of the authors and do not necessarily represent those of their affiliated organizations, or those of the publisher, the editors and the reviewers. Any product that may be evaluated in this article, or claim that may be made by its manufacturer, is not guaranteed or endorsed by the publisher.
