# Supplementary material for: Clinical Symptoms and Outcomes of Severe Pneumonia Caused by Chlamydia psittaci in Southwest China
Source: Front Cell Infect Microbiol. 2022 Jan 6;11:727594. doi: 10.3389/fcimb.2021.727594 (PMC8770948; doi:10.3389/fcimb.2021.727594)
Supplement: Supplementary file 1 [file DataSheet_1.pdf]

| patient | survivor | mNGS results and reads from BALF |
| --- | --- | --- |
| 1 | YES | Chlamydia psittaci （338） |
| 2 | NO | Chlamydia psittaci （7347） |
| 3 | YES | Chlamydia psittaci （14）,Candida glabrata (18) |
| 4 | YES | Chlamydia psittaci （72） |
| 5 | YES | Chlamydia psittaci （2880）,Candida albicans(5) |
| 6 | YES | Chlamydia psittaci （748） |
| 7 | YES | Chlamydia psittaci (1367) |
| 8 | YES | Chlamydia psittaci (75),Rothia aeria (30) |
| 9 | NO | Chlamydia psittaci (15234),Corynebacterium striatum (12) |
| 10 | YES | Chlamydia psittaci(7), Veillonellaparvula(26), Campylobacter conCisus(6) |
| 11 | YES | Chlamydia psittaci (1187),Corynebacterium striatum(8),Klebsiella Pneumoniae(22) |
| 12 | YES | Chlamydia psittaci (36),cytomegalovirus(5) |
| 13 | YES | Chlamydia psittaci (166),Streptococcus salivarius(12） |
| 14 | YES | Chlamydia psittaci (88) |
| 15 | YES | Chlamydia psittaci (777),herpes simplex virus-1(12) |
| 16 | YES | Chlamydia psittaci (150) |
| 17 | YES | Chlamydia psittaci (13) |
| 18 | YES | Chlamydia psittaci (27),candida Albicans（20） |
| 19 | YES | Chlamydia psittaci (152) |
| 20 | YES | Chlamydia psittaci (58)，Streptococcus pneumoniae（33） |
| 21 | YES | Chlamydia psittaci (4357)，Acinetobacter baumannii complex（363） |
| 22 | NO | Chlamydia psittaci (337) |
| 23 | YES | Chlamydia psittaci (1367) |
| 24 | YES | Chlamydia psittaci (353),Raoultella ornithinolytica (4) |
| 25 | YES | Chlamydia psittaci (5351),Corynebacterium striatum (4) ,Acinetobacter baumannii complex(4) |
| 26 | YES | Chlamydia psittaci (114),Rothia Geory and Brown(28) |
| 27 | NO | Chlamydia psittaci (1716),Haemophilus influenzae (5) |
